# Potent pollen gene regulation by DNA glycosylases in maize

**DOI:** 10.1101/2024.02.13.580204

**Published:** 2024-02-15

**Authors:** Yibing Zeng, Julian Somers, Harrison S. Bell, R. Kelly Dawe, John E. Fowler, Brad Nelms, Jonathan I. Gent

**Affiliations:** 1 University of Georgia, Department of Genetics, Athens, GA 30602; 2 Oregon State University, Department of Botany and Plant Pathology, Corvallis, OR 97331; 3 University of Georgia, Department of Plant Biology, Athens, GA 30602

**Keywords:** MDR1, DNA methylation, pollen vegetative cell, cell wall, Zea mays

## Abstract

Although DNA methylation primarily represses transposable elements (TEs) in plants, it also represses select endosperm and pollen genes. These genes, or their cis-regulatory elements, are methylated in plant body tissues but are demethylated by DNA glycosylases (DNGs) in endosperm and pollen, enabling their transcription. Activity of either one of two DNGs, MDR1 or DNG102, is essential for pollen viability in maize. Using single-pollen mRNA sequencing on pollen segregating mutations in both genes, we identified 58 candidate DNG target genes, whose expression is strongly decreased in double mutant pollen (124-fold decrease on average). These genes account for 11.1% of the wild-type pollen polyadenylated transcriptome, but they are silent or barely detectable in the plant body. They are unusual in their tendency to lack introns but even more so in their having TE-like methylation in their coding DNA sequence. Moreover, they are strongly enriched for predicted functions in cell wall modification. While some may support development of the pollen grain cell wall, expansins and pectinases in this set of genes suggest a function in cell wall loosening to support the rapid tip growth characteristic of pollen tubes as they carry the sperm cells through maternal apoplast and extracellular matrix of the pistil. These results suggest a critical role for DNA methylation and demethylation in regulating maize genes with potential for extremely high expression in pollen but constitutive silencing elsewhere.

## INTRODUCTION

In angiosperms, a single pollen grain is made up of a pollen vegetative cell that encapsulates two sperm cells. After pollen grain release from the anther and contact with a stigma, the pollen vegetative cell germinates into a pollen tube that rapidly elongates through the transmitting tract until it reaches the ovary and delivers one sperm to the egg cell to form the zygote and one to the central cell to form the endosperm (Hafidh and Honys 2021). Like unicellular root hairs, moss protonema, and fungal hyphae, the pollen tube elongates by tip growth. Also like fungal hyphae, it grows invasively, that is through cell walls and extracellular matrices (Adhikari et al. 2020). In doing so, it secretes proteins that loosen or modify cell walls, including expansins, pectinases, pectin methylesterases, and rapid alkalinization factors (RALFs) (Flores-Tornero et al. 2021). In theory these factors could act on the tube tip, on stigmatic epidermal cells, or on the extracellular matrix within the transmitting tract. Development of the pollen grain itself involves a complex process of building a multi-layered cell wall in coordination with the surrounding tapetal cells (Shi et al. 2015). In maize, the pollen tube is among the fastest growing eukaryotic cells and can reach a rate of 1 cm/hour as it travels through a style (silk) that can be 30 cm long (Miller 1919; Williams et al. 2016). In comparison, the rate of fast growing hyphae is on the order of 1.3 mm/hour (Jackson 2001).

The extreme growth rate of the pollen tube raises the possibility that the pollen transcriptome would be highly specialized. Indeed, pollen transcriptomes differ considerably from the transcriptomes of other plant tissues, appearing removed from others in multi-tissue analyses (Becker et al. 2014). While some transposable elements (TEs) are known to have enriched mRNA expression in pollen (Slotkin et al. 2009; Ibarra et al. 2012; Warman et al. 2020), repression of TEs is generally maintained in pollen in spite of increased chromatin accessibility because of multiple overlapping mechanisms of repression (He et al. 2019; Ichino et al. 2022). Some TEs are also hypothesized to be transcribed in the pollen vegetative nucleus in order to produce siRNAs that are transmitted to sperm nuclei to reinforce repression in the next generation (Slotkin et al. 2009; Ibarra et al. 2012; Martínez et al. 2016; Kim et al. 2019b). Consistent with robust TE repression in pollen, both sperm and vegetative nuclei have similar or higher DNA methylation levels than other cell types in Arabidopsis (Hsieh et al. 2016). There are notable locus-specific difference though, where the vegetative nucleus is demethylated relative to sperm. DNA demethylation occurs passively, through DNA replication, or actively, by specific replacement of methylated cytosines with unmethylated cytosines.

In plants, active DNA demethylation is accomplished by DNA glycosylases (DNGs), of the same type that function in base excision repair (Hemenway and Gehring 2023). These enzymes are essential for endosperm development in diverse angiosperms including Arabidopsis, rice, and maize, where they demethylate maternally imprinted genes (initiating demethylation in the central cell before fertilization (Choi et al. 2002; Park et al. 2016)). In addition, they demethylate thousands of other loci, most of which do not overlap genes at all (Ibarra et al. 2012; Rodrigues et al. 2021; Gent et al. 2022; Xu et al. 2022). The same DNGs that demethylate DNA in central cell and endosperm also demethylate overlapping sets of thousands of loci in the pollen vegetative nucleus, as evidenced by comparison of wild-type and mutant methylomes (Calarco et al. 2012; Ibarra et al. 2012; Kim et al. 2019b). In Arabidopsis, mutants of the DNG DEMETER have a weak and background specific defect in pollen tube growth, but double mutants lacking DEMETER and another DNG, ROS1, have a stronger phenotype in which pollen tubes growth is disoriented (Khouider et al. 2021). A DNG in rice, called DNG702 or ROS1A, is also important for pollen fertility and its mutant has earlier defects in microspore morphology (Zhou et al. 2021; Irshad et al. 2022). In Arabidopsis, 27 genes have been identified that are demethylated by DNGs in pollen and transcriptionally activated. Likely consistent with the disoriented pollen tube growth in DNG mutants, these genes are strongly enriched for kinases predicted to be involved in protein signaling (Borg et al. 2021; Khouider et al. 2021). Maize has four DNGs in three subtypes (Gent et al. 2022). The subtype that is highly expressed in endosperm has two paralogous genes, *mdr1* (also known as *dng101* (Jia et al. 2009) and *zmros1b* (Xu et al. 2022)) and *dng102*. Mutations in both *mdr1* and *dng102* can be transmitted through the maternal gametophyte simultaneously, but the resulting seeds abort early in development (GENT *et al.* 2022). Maternal transmission of either single mutant produces healthy seeds, as does paternal transmission. They cannot both be paternally transmitted together, however, indicating an essential function of these DNGs in the male gametophyte.

Understanding functions for DNA methylation in gene regulation in plants has proven difficult. Part of the difficulty is due to the complexity of DNA methylation and part to the complexity of genes themselves. For example, methylation that silences TEs located in introns has different effects than methylation located in cis regulatory elements. In cases where regulatory regions contain TEs or tandem repeats, it is difficult to distinguish genome defense mechanisms from normal developmentally or environmentally responsive gene regulation. The Arabidopsis genes *FWA* and *SDC* and the maize genes *b1* and *r1* provide a few of the many examples where gene regulation can be strongly affected by TE-related DNA methylation due to TEs or other repeats in their cis-regulatory elements (Walker 1998; Kinoshita et al. 2007; Henderson and Jacobsen 2008; Belele et al. 2013). Exons are frequently methylated in CG context alone, referred to as gene body methylation (Muyle et al. 2022). This is a common feature of broadly expressed genes, including in the cells where they are highly transcribed. TE-like methylation, where CG and CHG methylation together, can also occur in exons and is associated with transcriptional repression. CHH methylation, which is associated with RNA-directed DNA methylation in maize, can also be grouped under TE-like methylation but is negligible compared with CHG methylation.

In a recent survey of methylation patterns in maize genes, we identified a large set of genes with TE-like methylation, making up more than 10% of gene annotations across diverse maize genomes (Zeng et al. 2023). Closer inspection revealed that the vast majority of these are poorly expressed, not conserved even between cultivated maize stocks, and frequently overlap TE annotations. Intriguingly, a subset of the remaining genes with TE-like methylation are highly expressed in endosperm or in anthers and tassels (Zeng et al. 2023). Since tassels and anthers contain pollen, the subset of genes with both TE-like methylation and high expression in these tissues are good candidates for function in pollen, dependent on developmentally-specific demethylation. Together with the requirement for *mdr1* and *dng102* in pollen fertility, these observations led us to explore relationships between DNGs, genes that are repressed by TE-like methylation in the plant body, and pollen development.

## RESULTS

**Figure 1:**
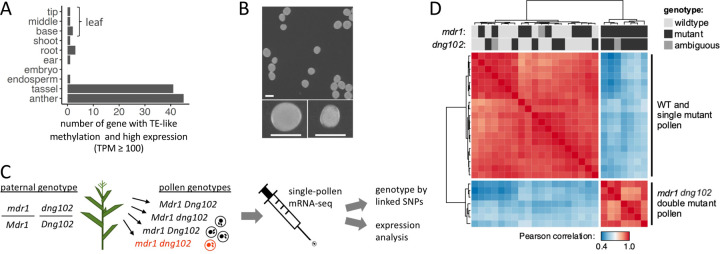
**(A)** Expression of genes with TE-like methylation in anther and tassel. The X axis indicates the number of genes in each of the ten tissues of Zeng et al (2023) which have TE-like methylation and TPM values of at least 100. This analysis only includes high confidence genes defined as genes that do not overlap with TE annotations and are part of the “core gene" set (Hufford et al. 2021). **(B)** Top panel, Pollen from an *mdr1/Mdr1 dng102/Dng102* a double heterozygous plant, segregating four haploid genotypes. Bottom panels show large and small pollen grains from the population above. Size bar = 100 mm. **(C)** Schematic of single-pollen mRNA-seq method. Individual libraries were prepared and sequenced for each pollen grain. Capital allele indicates WT, lowercase mutant, red font double mutant. **(D)** Unsupervised clustering of single-pollen transcriptomes based on Pearson correlation across the entire dataset (all x all). Warmer colors indicate stronger correlations between transcriptomes. The top two rows indicate *mdr1* and *dng102* genotypes inferred from SNPs linked to the two loci derived from RNA-seq data, which were scored independently of the transcriptome correlation analysis. Genotypes: black is mutant, light grey is wild-type, and dark grey is ambiguous.

To better quantify the number of expressed, non-TE genes with TE-like methylation, we enriched for high-confidence gene annotations by including only those encoded at syntenic positions in B73 and 25 other diverse maize genomes that are founders for the Nested Association Mapping (NAM) population (i.e., part of the defined core gene set (Hufford et al. 2021)) and whose coding DNA sequence (CDS) did not overlap with TE annotations. For each of the ten diverse tissues assessed by RNA-seq in the prior study (Hufford et al. 2021), we counted the number of these high-confidence genes with TE-like methylation expressed at increasing TPM thresholds (Figure 1A and Supplemental Figure 1). The two pollen-containing tissues, anther and tassel, clearly stood out. With a moderate threshold (TPM ≥ 100), 45 genes with TE-like methylation were expressed in anther and 41 in tassel (37 overlapped). No other tissue had more than three expressed genes with TPMs of at least 100, and none overlapped with the 45 in anther.

These results pointed toward pollen as a key tissue with active expression of genes with TE-like methylation, potentially targeted by DNGs for tissue-specific demethylation. We established previously that *mdr1* and *dng102* loss-of-function mutants could not be transmitted together through pollen (< 0.005% transmission; GENT *et al.* 2022), indicating that these two genes are redundantly essential in pollen. To evaluate the function of MDR1 and DNG102 we visually examined pollen from plants that were heterozygous for both *mdr1* and *dng102* mutations, to assess whether any strong morphological defect would show up in ¼ of the haploid pollen (Figure 1B and C). Although there was no conspicuous increase in qualitative morphological defects in these populations of pollen, quantitative analysis of two-dimensional pollen area from microscope images revealed a bimodal distribution of pollen from double mutant, but not single mutant or wild-type plants, i.e., segregation of a small pollen (sp) phenotype (Supplemental Figure 2A). The size of the secondary peak of pollen areas was consistent with an sp phenotype in ¼ of the pollen and a ~35% reduction in area, corresponding to a ~50% reduction in volume. Moreover, the sp phenotype was also present in pollen populations derived from plants carrying a second, independent *mdr1* allele alongside the *dng102* mutant, co-segregating in sibling plants with the parental double mutant heterozygous genotype (Supplemental Figure 2B).

We next sought to determine how gene expression was affected in the *mdr1 dng102* double mutant pollen. The inability to generate plants homozygous for both *mdr1* and *dng102* makes traditional bulk expression analysis infeasible; we overcame this by directly sequencing RNA from individual pollen grains (Nelms and Walbot 2022; Washburn et al. 2023), making it possible to compare double mutant to single mutants and wild-type sibling pollen grains (Figure 1C). We obtained transcriptomes from 26 individual pollen grains collected from an *mdr1/Mdr1 dng102/Dng102* double heterozygous plant, detecting a mean of 549,559 mRNA transcripts (Unique Molecular Identifiers; UMIs) and 9,396 expressed genes per pollen grain.

To determine the individual pollen genotypes, we reasoned that expressed SNPs linked to *mdr1* and *dng102* would allow us to infer the genotype of each pollen grain directly from its transcriptome. Mutant alleles for both genes were originally isolated in a B73 stock but then back-crossed into W22 for five generations (Gent et al. 2022); as a result, these plants were predominantly W22 but had a several Mb region of B73 sequence surrounding each mutant allele. We analyzed SNPs in the single pollen RNA-seq data to determine whether transcripts for genes neighboring *mdr1* and *dng102* were from the W22 or B73 alleles (*mdr1* and *dng102* were expressed at too low a level to genotype directly). Genotypes were assigned for both *mdr1* and *dng102* in 23 of 26 pollen grains (Supplemental Figure 3); the remaining 3 pollen grains were ambiguous for either *mdr1* or *dng102* due to recombination between the linked genes used for genotyping (Supplemental Figure 3E and 3F). In total, we found 4, 7, 6, and 6 pollen grains with the *Mdr1 Dng102*, *mdr1 Dng102*, *Mdr1 dng102*, and *mdr1 dng102* genotypes, respectively, consistent with expectations for random segregation of both mutant alleles (p = 0.843; chi-squared test).

**Figure 2:**
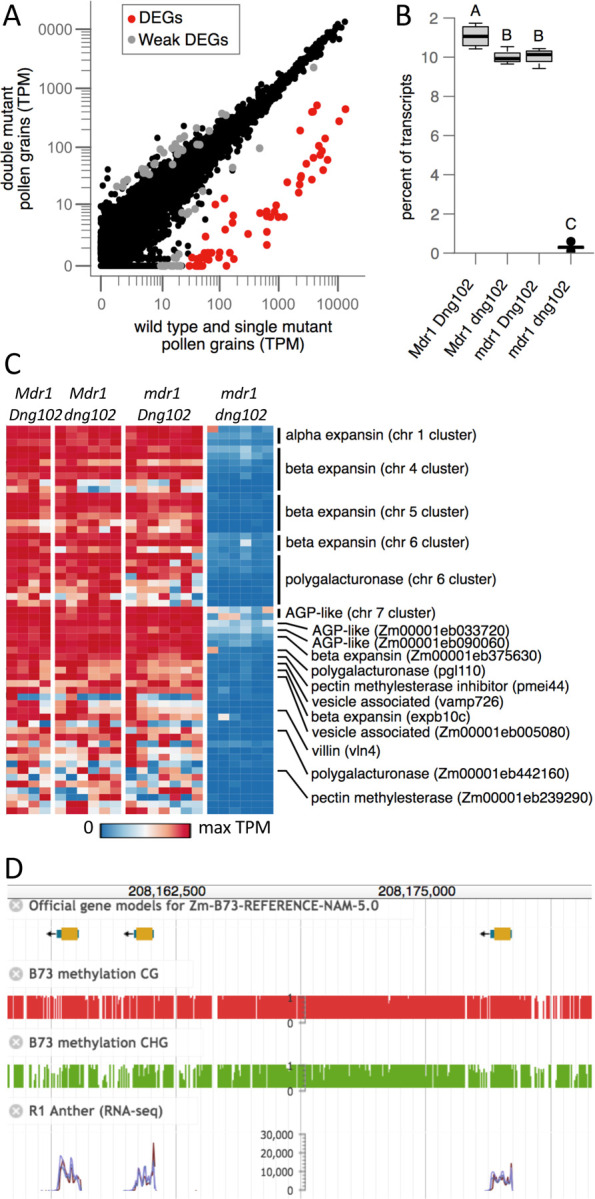
**(A)** WT and single mutant vs. double mutant gene expression. Dots correspond to individual genes. Axes indicate mean TPM values for each set of transcriptomes. The strongly differentially expressed genes (DEGs, red dots) have ≥ 8-fold change in expression in double mutant and an average of ≥ 10 UMIs in the WT and single mutant. An additional 48 genes (Weak DEGs, grey dots) showed evidence of differential expression by less stringent criteria (adjusted p-value ≤ 0.05; ≥ 2-fold change in expression). **(B)** Total expression of the 58 DEGs as percent of all measured transcripts. Boxplots indicate the median (horizontal dark line), interquartile range (box), and range (vertical lines) of the measured values. Letters indicate statistically significance: groups not sharing a letter have a significantly different mean (p ≤ 0.05; Tukey’s Honest Significant Difference Test). **(C)** Expression patterns of DEGs in each pollen grain. Each row represents a single gene, and each column a pollen grain, organized by genotype. Rows are sorted by chromosome, position, and TPM, with genes in clusters listed above singletons. **(D)** MaizeGDB browser image of an approximately 30 Kb part of a beta expansin gene cluster on chromosome 5. Included are publicly available DNA methylation tracks and anther gene expression tracks (Hufford et al. 2021).

Unsupervised hierarchical clustering of the single-pollen transcriptome data produced two distinct clusters, one with 19 pollen grains and one with 7, (Figure 1D). These clusters perfectly separated pollen with the double mutant *mdr1 dng102* genotype from all others (Figure 1D), showing that there was a strong and reproducible gene expression change in the double mutant. In contrast, there was no separation of *mdr1* or *dng102* single mutant pollen grains from wild-type or from each other, indicating relatively less transcriptional change in the single mutants. To identify genes that were mis-expressed in the double mutant pollen, we used DESeq2 to assess differential expression relative to wild-type and single mutant pollen grains. One-hundred and six genes were differentially expressed with moderate cutoffs (adjusted p-value ≤ 0.05; ≥ 2 fold change in expression), with 58 exceeding very strong criteria for differential expression (≥ 8-fold change in expression; mean UMIs ≥ 10). All 58 of these strongly differentially expressed genes (DEGs) were downregulated in the double mutant pollen, with a median decrease of 124.1-fold (Figure 2A). The 58 DEGs made up 11.1% of all detected mRNA transcripts in WT pollen (Figure 2B), representing some of the most highly expressed genes. In contrast, these genes made up only 0.3% of transcripts in the double mutant. This is consistent with a model where MDR1 and DNG102 are required to demethylate a set of strong pollen-expressed genes so that they can be properly expressed. There was also a mild reduction in the expression of the DEGs in both *mdr1* and *dng102* single mutant pollen grains, suggesting a slight expression defect in the single mutants; however, these changes were too weak to detect without knowledge of the potential target genes (Figure 2B and Supplemental Figure 4), and so it is unsurprising that both single mutants can be readily transmitted through pollen while the double mutant cannot.

Many of the DEGs shared similar genomic features. Half (28 genes) occurred in six clusters of two to eight differentially expressed copies (Supplemental Table 1 and Figure 2C and D). The clusters were not simple head-to-tail gene arrays, but included variable amounts of DNA between genes. Some of the clusters also carried additional gene copies that were not detected as DEGs. There was also a strong trend for the DEGs to have only one or two exons. Of the genes in clusters, 27 were annotated with a single exon in the canonical transcript and one with two exons. Of the other 30 DEGs, ten have single exons and five have two exons.

37 of the 58 DEGs can be linked to cell wall functions. Four of the six clusters encode expansins (one alpha type, four beta types), and one encodes polygalacturonases (pectinases). When secreted into the apoplast, expansins and pectinases loosen cell walls (Kende et al. 2004; Yang et al. 2018). The sixth cluster encodes two uncharacterized proteins of 69 and 76 amino acids with homologs across the grass family (Supplemental Figure 5). The closest matches to these proteins in Arabidopsis are the arabinogalactan protein AGP11 and its homolog APG6, with the two maize AGP-like proteins showing 23% aa identity match to the first 74 aa of the 136-aa of AGP11. Although the molecular functions of AGP11 and AGP6 are unknown, but they localize to cell walls, and inhibiting their function is associated with defects in the nexine layer of the pollen grain wall and in pollen tube growth (Costa et al. 2013; Jia et al. 2015). The 30 singleton DEGs were also enriched for predicted functions in cell walls. These include two beta expansins, two polygalacturonases, one pectin methylesterase inhibitor and one pectin methylesterase. The pectin methylesterase is part of another cluster of multicopy genes that has a role in overcoming maternal barriers to fertilization as part of the Ga2 unilateral cross incompatibility system (Chen et al. 2022). The highest expressed of the DEGs were two more AGP-like unlinked paralogs encoding 70-aa and a 72-aa proteins with about 26% aa identity with the two clustered AGP-like DEGs and about 29% aa identity with the first 74-aa of the Arabidopsis AGP11 protein (Supplemental Figure 5). Two other DEGs were paralogs encoding vesicle associated membrane proteins, one of which, VAMP726, has been shown to influence lignin content in the maize pollen cell wall (Yang et al. 2023). In total, 39 of the 58 DEGs are predicted to have cell wall related functions, and 32 of those are expansins and proteins related to pectin degradation or modification, likely involved with pollen tube growth. Another gene with potential function in rapid growth is an actin-binding villin protein. The remaining 17 DEGs did not show any clear trends in terms of predicted function.

**Figure 3:**
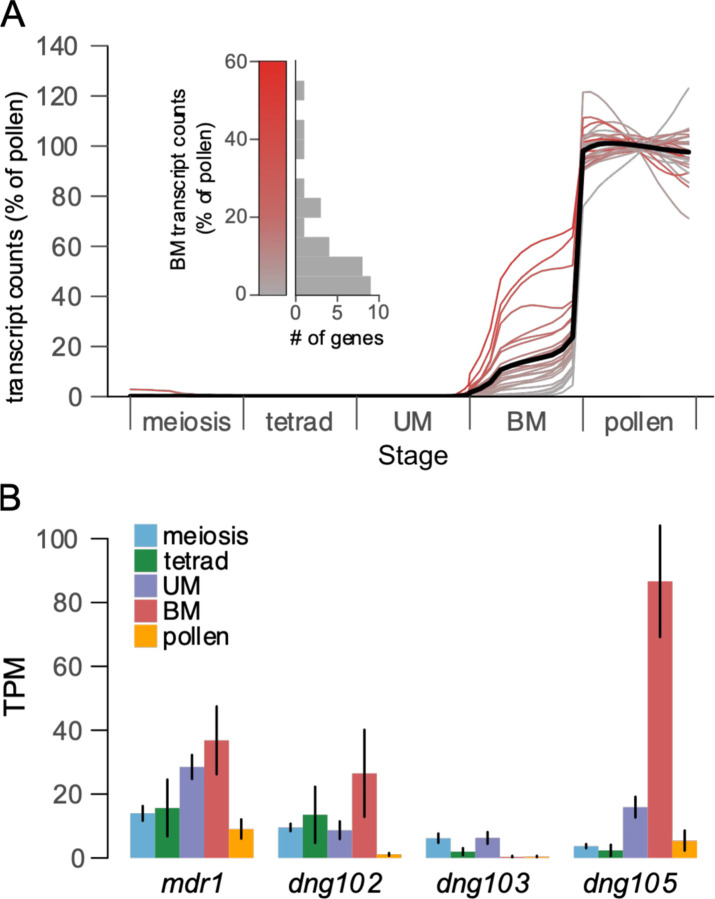
**(A)** Expression timecourse of DEGs. The bold line represents the average expression profile across all DEGs. UM = unicellular microspore, BM = bicellular microspore. Data are from B73/A188 hybrids, normalized to the mean transcript abundance in pollen (Nelms and Walbot 2022). The timecourse spans 349 pollen grains and precursors, here reported as a rolling weighted average by pollen precursor stage (see methods); a heatmap without any averaging is visible as Supplemental Figure 6. The variation seen in mature pollen for two genes in particular is consistent with random noise and not statistically significant. Grey to red color scale indicates expression level at bicellular stage, as quantified in the inset. **(B)** Expression timecourse of the four maize DNGs *mdr1, dng102, dng103,* and *dng105* using the same data as in (A). Low expression of these genes makes them unsuitable for the graphical representation used in (A). Instead, bar heights indicate average TPM values from individual pollen and pollen precursors, and error bars indicate standard errors.

To determine when the DEGs were first expressed during pollen development, we examined an expression timecourse that covers the beginning of meiosis through mature pollen (Nelms and Walbot 2022). The DEGs showed undetectable or very little expression during meiosis and early haploid stages, but were then up-regulated to varying degrees at pollen mitosis I (Figure 3A and Supplemental Figure 6) corresponding to the major wave of haploid (gametophyte) genome activation in maize (Nelms and Walbot 2022). This coincides with peak expression of *mdr1* and *dng102* (Figure 3B), but unlike their potential targets, both DNGs were also detectably expressed throughout meiosis and early pollen development. Altogether, this suggests that MDR1 and DNG102 act on their target genes sometime before or shortly after pollen mitosis I. While the earliest-expressed DEGs were up-regulated at pollen mitosis I, others were not strongly expressed until the mature pollen stage. This might suggest a second wave of MDR1/DNG102 activity, but could also be explained by a single, earlier period of DNA demethylation followed by multiple waves of transcription for these genes.

**Figure 4:**
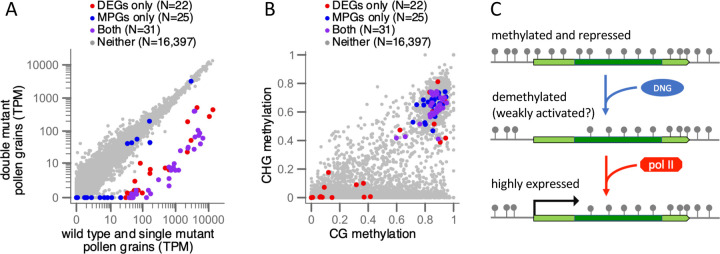
**(A)** The TE-like methylation character mapped onto the differential expression analysis comparing WT and single mutant vs. double mutant gene expression. Axes indicate the mean TPM values for each set of transcriptomes. Methylated pollen genes (MPGs) have TE-like methylation and at least tenfold higher expression in anther than in eight vegetative tissues in B73. Only core genes that are annotated in the W22 genome and in all 26 of the NAM founder genomes and which have sufficient coverage of EM-seq reads were included in this analysis. **(B)** CG methylation vs. CHG methylation for the same sets of genes displayed in (A). Methylation values are measured in coding DNA sequence only, as a proportion of methylated cytosines to total cytosines, and range from 0 to 1. **(C)** A model for DNA methylation in pollen gene regulation, requiring an initial licensing step by DNA glycosylases (DNGs) removing methylation (gray lollipops), before transcription at high levels via gene-specific transcription factors (not shown) recruiting RNA polymerase II.

Since a major motivation for this study was our observation that a set of genes with TE-like methylation has high expression in pollen containing tissues, we asked whether that set overlaps with the candidate DNG target genes we identified as DEGS in the *mdr1* and *dng102* mutant pollen. To answer this quantitively, we identified genes with TE-like methylation and syntenic conservation in maize and that had at least tenfold more expression in anthers than in the eight other non-pollen-containing plant tissues accessed by RNA-seq in the same study. This produced a set of 56 genes, which we refer to as methylated pollen genes (MPGs) for brevity. While we required only a tenfold increase in gene expression relative to each of the other eight tissues, the average fold change for MPGs was actually far greater because MPGs were either undetectable or barely detectable in these tissues. The MPGs, were identified with methylation and expression data from a B73 stock yet strongly overlapped (36 of 56) with the independently-identified DEGs from a W22 stock (Figure 4A and B). The genetic differences between W22 and B73would be expected to reduce the amount of overlap between gene sets. Consistent with this, an additional 12 MPGs were in the same seven clusters as DEGs, and three more were unlinked paralogs of DEGs (Supplemental Table 1). Forty-five of these 51 MPGs had predicted functions in cell wall modification. The five MPGs that were neither in clusters nor paralogs of DEGs encoded an RNA binding protein; an oxalidate oxidase; two WEB domain paralogs implicated in actin-mediated plastid movement (Suetsugu and Wada 2016); and most striking because of its high expression in pollen, RALF1, a member of the Rapid Alkalinization Factor family of secreted small proteins that influence the cell wall via interaction with receptors and other apoplastic molecules (Flores-Tornero et al. 2021; Zhou et al. 2023).

Assuming the stable expression of some MPGs like *ralf1* in *mdr1 dng102* double mutant pollen is biologically meaningful and not a technical artifact of comparing data derived from W22 vs. B73, a theoretical explanation is that the more distantly related DNG, *dng105* (Gent et al. 2022), is also expressed in pollen and pollen precursors (Figure 3B). In the absence of MDR1 and DNG102, DNG105 might act redundantly on a subset of genes to activate gene expression. Conversely, DEGs with little or no TE-like methylation in their coding DNA (Figure 4B) could be explained by indirect effects of demethylation of other genes or by demethylation of their cis regulatory elements rather than coding DNA. Regardless, the clear pattern is that genes with TE-like methylation and high expression in pollen tend to be dependent on the DNGs MDR1 and DNG102.

## DISCUSSION

Two different approaches converged on similar sets of genes—especially genes that are highly expressed in pollen, encoded in one or two exons, and are predicted to modify cell walls. One approach relied on differential gene expression in DNA glycosylase mutants known to be essential for pollen function. The other identified genes based on TE-like DNA methylation in leaf and on anther-specific gene expression. Combined with prior evidence for DNGs in the pollen vegetative nucleus driving gene expression needed for pollen fertility in Arabidopsis and rice, these results indicate that DNG-mediated gene regulation in pollen is widely conserved in angiosperms. While the form of gene regulation is broadly conserved, the specific genes differ. The data from Arabidopsis DNG mutants suggest an enrichment for genes involved in cell signaling controlling orientation of growth (Borg et al. 2021; Khouider et al. 2021) though an earlier study in Arabidopsis also noted an enrichment for genes with TE-like methylation involved in modifying cell walls in pollen (Schmitz et al. 2013). (That study used the term “RdDM targets” as the term “TE-like methylation” had not been adopted yet). These differences in target genes between the two species may explain differences in phenotypic effects caused by loss of DNG function—complete pollen infertility in maize vs. a mildly reduced transmission (with aberrant pollen tube growth orientation and some reduced pollen tube germination) in Arabidopsis (Schoft et al. 2011; Khouider et al. 2021). In maize, a primarily outcrossing species with an extensive stigma for pollen reception and pollen in excess, rapid pollen tube germination and growth is critical for successful competition and eventual fertilization. Although the expansins, pectinases, and pectin methylesterases regulated by DNGs in maize pollen predict a pollen tube growth defect in *dng* mutants, an earlier pollen defect might prevent a tube phenotype from ever manifesting. In theory, lack of a single DNG target could lead to a small pollen phenotype with a large impact on pollen fertility, similar to *sp1*, *sp2*, and *stt1* mutants (Rhoades and Rhoades 1939; Singleton and Mangelsdorf 1940; Phillips and Evans 2011).

Maize DNGs, including *mdr1* and *dng102*, are expressed in other cell types, particularly endosperm. There are notable differences between the DNG target genes we identified in pollen and ones already identified in endosperm. In diverse angiosperms, key endosperm genes that are upregulated by DNGs (specifically from the maternal genome), function in gene regulation themselves, producing cascading effects on gene expression. These genes include members of the polycomb repressive complex PRC2 and ethylene signaling pathways that have central roles in early endosperm development (Gehring et al. 2006; Haun et al. 2007; Hermon et al. 2007; Tonosaki et al. 2021; Ando et al. 2023). Thus, moderate expression of these genes initiates a cascade of gene expression changes indirectly resulting from DNA demethylation. In contrast, the DNG target genes in pollen appear to be massively upregulated directly. In endosperm, DNG target genes exhibit a strong tendency for methylation in their promoters and 5’ UTRs rather than coding exons (Gent et al. 2022), whereas the pollen genes are methylated not just in promoters and 5’ UTRs but also across CDS. In fact, our observation of methylation in CDS partially motivated this study (Zeng et al. 2023).

In some animals, DNA methylation in promoters functions with other chromatin modifications as a developmentally stable form of transcriptional repression. This occurs in the repression of germline genes in somatic cell lineages and across the X chromosome in X inactivation (Greenberg and Bourc'his 2019). Although though are there are many examples of repetitive elements acting as cis regulatory elements that sensitize gene expression to DNA methylation, such as the Arabidopsis *FWA* and *SDC* genes and the maize *r1* and *b1* genes (Walker 1998; Kinoshita et al. 2007; Henderson and Jacobsen 2008; Belele et al. 2013), DNA methylation is not a common aspect of developmental gene regulation in plants. Rather, as well established in maize, cis regulatory elements remain constitutively free of methylation, regardless of which cells the genes are expressed in (Crisp et al. 2020; Hufford et al. 2021). This is also true of their coding DNA, except in CG context (Zeng et al. 2023). Keeping cis regulatory elements free of methylation may be a major function of DNGs in the plant body, allowing dynamic access of both activating and repressing factors (Halter et al. 2021). Transcription factors provide both tissue specificity and sequence specificity to repression by recruiting histone modifiers such as the polycomb repressive complexes PRC1 and PRC2, which ubiquitinate histone H2A at lysine 119 (H2AK119ub) and methylate histone H3 lysine 27 (H3K27me3) (Baile et al. 2022; Strader et al. 2022).

The vast majority of genes in the pollen vegetative nucleus are likely regulated using the same mechanisms as other plant cells since they are neither differentially expressed in *mdr1 dng102* double mutant pollen nor have TE-like methylation in the plant body. We hypothesize that the highly expressed DNG target genes we identified require a two-step activation in pollen—first, recruitment of DNGs to create an environment permissive for RNA polymerase, followed by recruitment of high levels of RNA polymerase itself (Figure 4C). As in endosperm, unidentified factors would recruit DNGs to some methylated loci and not others. Transcription factors, because of their roles in guiding protein complexes to specific loci would be good candidates. This two-step activation using DNA methylation as the basis for repression could allow for the huge dynamic range of expression we observe, from nearly undetectable in most cells to 11% of the transcriptome in pollen.

Why not use DNA methylation in gene regulation more broadly? One possibility is that unique epigenetic features of the pollen vegetative nucleus make it better suited to this form of gene regulation (He et al. 2019; Ichino et al. 2022).. Another reason for limiting the use of DNA demethylation in gene activation could be an elevated risk of mutation associated with excising methylated cytosines.. Regardless, these results point to a role for DNA demethylation in potent and cell type-specific gene regulation in the pollen vegetative nucleus. This form of regulation not only allows for massive upregulation of gene expression in pollen, but also for robust silencing outside of pollen.

## METHODS

### Pollen phenotyping

100 to 600 uL freshly shed pollen was collected in tassel bags before being added to individual microcentrifuge tubes containing 800 uL EAA fixative solution (3:1 ethanol to acetic acid). Pollen samples were inverted 3x before being parafilmed and stored at 20 C. Samples were imaged using a LEICA M205 FCA Fluorescence stereo microscope and the Leica Application Suite X (V 3.7.5.24914). Resulting images were imported into Fiji (ImageJ) (V 2.0.0) and subjected to the following processing pipeline: ‘Image -> Enhance Contrast’ (0.3%), ‘Adjust - >Threshold Image’ (Auto -> Apply), ‘Process -> Binary -> Make Binary’, ’Process -> Binary -> Watershed’, ‘Analyze -> Analyze Particles (Size (micron^2): 2000–14000, Circularity: 0.75–1.00, Show:Nothing, Display Results, Summarize, Exclude on edges, Include holes) -> Okay’. Particle measurements copied into a .csv file and loaded into R (V. 4.2.3) to produce plots with ggplot2 (V 3.4.3) and ggridges (0.5.4) packages.

### Analysis of DNA methylation and expression data of B73 genes

To identify genes with TE-like methylation, we reanalyzed methylation data from developing leaf for the maize B73 genome (Hufford et al. 2021) using the same methods as in our recent study of methylation in genes (Zeng et al. 2023). This method only makes use of methylation within annotated CDS, as introns often have TE-like methylation for the simple reason that they contain introns, and UTRs are difficult to annotate accurately. To include more genes with short CDSs, for this study we required only 30 individual informative CGs and individual informative 30 CHGs per gene rather 40 of each. “Informative” means they spanned by at least one EM-seq read where the C in the genome could unambiguously be associated with either a C or a T in the read. As before, genes with average methylation levels of at least 40% in both CG and CHG methylation were defined as having TE-like methylation. We only included the core gene set in these analyses, the ones that are present at syntenic positions in B73 and the 25 other NAM founder genomes, as defined previously (Hufford et al. 2021). This yielded 926 genes with TE-like methylation (teM), 7882 with gene body methylation (gbM), 13098 that were unmethylated (UM), 3661 that had intermediate methylation values (ambiguous), and 2724 that did not meet the requirements for sufficient informative cytosines. These methylation epialleles are listed along with gene names and methylation values in a table available on Github (https://raw.githubusercontent.com/dawelab/MethylatedPollenGenes/main/Data/df_RedefineEpailele.csv?token=GHSAT0AAAAAACMFLIBUHCBROGBSM7N7KK6AZNKUPWQ).

To more meaningfully quantify expression of genes with TE-like methylation in the ten tissues, we further enriched for functional genes by excluding all gene annotations whose CDS overlapped with annotated TEs. Gene and TE annotations were the same as the ones used in the prior study, obtained from https://download.maizegdb.org/Zm-B73-REFERENCE-NAM-5.0/. Using a combination of unix cut, awk and sed commands, we converted the source gff3 gene annotation file ZM-B73-REFERENCE-NAM-5.0_Zm00001eb.1.ggf3 into bed format with a geneID for each CDS region. We then used the BEDTools v2.30.0 (Quinlan and Hall 2010) intersect tool to identify all genes whose CDS overlapped with TEs in Zm-B73-REFERENCE-NAM-5.0.TE.gff3 by even a single base. Then we used awk to select the geneID columns and uniq to remove redundant rows corresponding to different CDSs from the same gene. We imported the geneID with TE-overlapping CDSs into an R data frame with row names as genes and a second column indicating TE overlaps by a value of 1. Then we merged this dataframe with a list of all core B73 genes using the R merge functionto create a new data frame where genes whose CDS did not overlap TEs had “NA” in the second column. Finally, we replaced all NA values in the second column with 0 and used this to filter each subset of genes to remove ones with TE-overlapping CDSs using the R tidyverse filterfunction. This yielded 394 teM genes, 6544 gbM genes, 11873 UM genes, 3975 that had intermediate values (ambiguous genes), and 2383 that did not meet the requirements for sufficient informative cytosines. These methylation epialleles are also listed along with gene names and methylation values in the table above.

To count the numbers of genes that expressed at or above different TPM thresholds in each tissue, we used the TPM matrix produced in the prior study (Zeng et al. 2023)(https://raw.githubusercontent.com/dawelab/Natural-methylation-epialleles-correlate-with-gene-expression-in-maize/main/Data/B73.all.csv). We used the R merge function to combine dataframes containing the methylation epiallele information for the core genes that did not have TE-overlapping CDSs with their TPM values in each tissue. Then we obtained counts of expressed genes in each tissue at each TPM threshold using the R group_by function to process each tissue separately followed by the summarise function then sum functions. We then used a for loop in R to iterate over a series of TPM thresholds from 1 to 100.

### Sequence comparisons of arabinogalactan pollen proteins

We used NCBI blastp to identify best homologs of the AGP-like proteins in sorghum, rice, and wheat using default parameters with the “Non-redundant protein sequences (nr)” as Database, “grass family (taxid:4479)” as Organism and sequences of Zm00001eb316010_P001 and Zm00001eb033720_P001 as Query. The resulting GenBank accessions of the best matches were used for sequence comparisons: KAG0544127.1 (Sorghum bicolor), EAZ09485.1 (Oryza sativa), XP_044385131.1 (Triticum aestivum), KAG0524731.1 (Sorghum bicolor), ATS17269.1 (Oryza sativa), and XP_044386294.1 (Triticum aestivum). We used Geneious^®^ 10.1.2 Tree Builder for protein tree construction using global alignment with free end gaps, identity cost matrix, Jukes-Cantor Genetic distance model, UPGMA method, gap open penalty of 6, and gap extension penalty of 3. The three Arabidopsis thaliana proteins we included in the tree are AT3G01700.1 (AGP11), AT5G14380.1, (AGP6) and AT5G64310.1 (AGP1). For pairwise comparisons between maize proteins and AGP11, we obtained amino acid identities using Geneious^®^ 10.1.2 global alignment with free end gaps, cost matrix identity, gap open penalty of 12, and gap extension penalty of 3.Zm00001eb316010 was 23% identical to AGP11, Zm00001eb033720 was 29% identical to AGP11, and Zm00001eb316010 and Zm00001eb033720 were 26% identical to each other.

### Single-pollen mRNA sequencing

For plant material, the *EMS4–06835d* allele of *mdr1* and the *dng102-Q235* allele of *dng102* were used (Gent et al. 2022). Both alleles originated in B73 stock and had been backcrossed for five generations into W22 (*mdr1* as stock J657 and the *dng102* stock as stock J658). Both stocks were then crossed together to create the double heterozygous stock *EMS4–06835d/Mdr1* and *dng102-q235/Dng102*, which were planted in late spring 2022 in a greenhouse in Athens, GA and grown under ambient light conditions until pollen collection in July.

Single pollen isolation and RNA-seq library prep were performed as described previously (Washburn et al. 2023). Briefly, pollen were released from anthers into a drop of 0.1X PBS by cutting transversely with a scalpel. Individual pollen grains were then manually isolated with a syringe needle and placed on the cap of a PCR 8-tube strip. RNA-seq libraries were then prepared with CEL-Seq (Washburn et al. 2023). Oligos for first-strand cDNA synthesis are available in Supplemental Table 2 (this replaced the “Barcoded CEL-seq primer plate” in Washburn et al. 2023); all other oligos and reagents are as described.

Sequencing data were analyzed similar to Nelms and Walbot (2022). Read 2 of these CEL-seq libraries contain a 10 nucleotide (nt) Unique Molecular Identifier (UMIs; https://doi.org/10.1101/gr.209601.116), followed by a 6 nt sample-specific barcode, and then a long string of Ts originally from the mRNA polyA tail; read 1 contains sequence matching the mRNA transcript. Paired-end reads were first demultiplexed based on sample-specific barcodes in read 2, requiring a perfect match to one of the expected barcode sequences (Supplemental Table 2). UMIs were then extracted from read 2 and appended to the read 1 sequence identifiers. No more information was used from read 2 and the remainder of analysis was on read 1 only.

Prior to mapping, reads were trimmed and filtered using Fastp v0.23.2 (Chen et al. 2018) with parameters -y -x -3 -a AAAAAAAAAAAA. Then filtered reads were mapped to the B73 v5 genome (ref) using Hisat2 v2.2.1 (Kim et al. 2019a) with the parameter -dta. De novo transcript assembly was then performed using Stringtie v2.2.1 (Kovaka et al. 2019), guided by the reference gene annotations and using the strand-specific information available from CEL-seq (parameter ‘-rf’; CEL-seq libraries map specifically to the coding strand). Features were assigned to the mapped reads (e.g. gene IDs) using featureCounts v1.2.5 and parameters -s 1 - readExtension5 500. Then unique transcripts were counted using the umi_tools v1.1.2 (Smith et al. 2017) ‘count’ function with parameter –per-gene. For pollen grain genotyping (Supplemental Figure 3), mapping was performed as above but using the W22 v2 reference genome (Springer et al. 2018); the reason for this difference was purely historical: we initially aligned to W22, but found that it was easier to work with B73 annotations because of greater consistency with other datasets.

### Quality control for RNA-seq samples

In total, 48 pollen grains were collected and sequenced. The resulting libraries showed two clear populations with varying library complexity. Twenty-seven pollen grains had high read depth, with a mean of 548,866 UMIs detected per pollen grain (range: 192,456 to 866,263). The remaining twenty-one pollen grains had much lower read depth, with a mean of 5,444 UMIs detected per pollen grain (range: 2,969 to 17,197). These two populations showed no enrichment for a given genotype or sample batch; a likely explanation is that some of the pollen grains failed to lyse completely, resulting in low library complexity. The 21 pollen grains with low complexity were excluded from further analysis.

One additional pollen grain was excluded because it showed several anomalous behaviors. First, it had relatively low correlation with every other pollen grain in the dataset. Second, when genotyping the genes near *mdr1*, there was a consistent trend for expression from both the B73 and W22 alleles; there was no other sample with noticeable biallelic expression, and this trend was not true for any genes near *dng102*. The conclusions of the paper were not sensitive to the inclusion or exclusion of this one pollen grain, but given the anomalies in the data, it was excluded.

### Analysis of single-pollen RNA-seq data

To genotype individual pollen grains, mapped RNA-seq data were visualized using the Integrative Genomics Viewer (IGV) (Thorvaldsdóttir et al. 2013). Three “sentinel” genes were selected on both sides of both the *mdr1* and *dng102* genes (12 genes in total), based on availability of mapped RNA transcripts with SNPs that distinguished the B73 vs W22 alleles. The mapped data were visualized to assign each sentinel gene to the B73 or W22 alleles. The B73 alleles are linked to the mutant alleles of *mdr1* and *dng102* while the W22 alleles are linked to wild-type. Some positions were scored as ambiguous if there were not reads spanning the SNPs that distinguish B73 and W22. The sentinel genes were then used to infer the alleles of *mdr1* and *dng102*, requiring consistency in allele calls on both sides (Supplementary Figure 3).

For the correlation heatmap in Figure 1D, the expression count matrix was normalized to transcripts per million (TPM) and then log-transformed after adding a pseudocount of 1. Genes with a mean expression under 500 TPM were filtered, and then the pairwise Pearson’s correlation was calculated between all samples.

Differential gene expression analysis was performed using DESeq2 (Love et al. 2014) with default parameters. Unadjusted p-values were then adjusted for multiple hypothesis testing using Holm’s method. Significant genes were identified as follows: for the “DEG” set, we required an adjusted p-value ≤ 0.05, an estimated log2 fold change ≥3, and a baseMean ≥10; for the “weak DEG” set, we required an adjusted p-value ≤ 0.05 and a log2 fold change ≥1.

### Timecourse of gene expression during pollen development

For Figure 3, the mapped transcript count matrix from Nelms and Walbot (2022) and associated sample metadata (e.g., the developmental stage of each sample) was downloaded from the Gene Expression Omnibus (accession GSE175505). These data, from a B73/A188 hybrid, were mapped to the B73 v4 maize genome, and so we determined the v4 IDs of the strong DEGs using the maizeGDB “Translate Gene Model IDs” tool; 41 of 58 DEGs had an associated v4 ID (Supplemental Table 3). We further excluded 12 genes that had very low expression in pollen in the 2022 dataset (< 10 TPM), as these may result from incorrect mapping. This left 29 DEGs that were analyzed for their timing during pollen development.

There are large changes in the total number of mRNA transcripts per pollen grain or precursor at different stages of pollen development, and so normalization methods that assume a constant total transcriptome size can be misleading (Nelms and Walbot 2022). For example, a gene with the same number of transcripts in a nearly-quiescent cell and in a transcriptionally active cell would highly appear to be differentially expressed by conventional TPM measurements. Thus, to better determine both the timing and level of DNG target expression, we used a normalization strategy that accounted for the differences in total transcript abundance between pollen and each pollen precursor stage. The data was first normalized to transcripts per million (TPM), but then scaled based on the fraction of absolute transcripts detected at a given stage relative to pollen. For instance, a mean of 133,905 and 377,873 UMIs was detected per individual BM stage precursor and mature pollen grain, respectively. Thus, all of the TPM-normalized data for BM stage precursors was scaled by 35.4% (133905 / 377873), preserving the relative difference in total transcripts between BM and pollen. The main effect of this choice on our conclusions is that all DEGs were expressed at a lower level in BM than Pollen (Figure 3), while if using TPM normalization there were 3 genes with higher expression in BM than Pollen. Thus, the TPM normalization might lead to the misleading conclusion that these 3 genes were downregulated between BM and pollen, even though the data is most consistent a situation where these 3 genes continue to increase in transcript abundance between BM and pollen, but at a lower rate than many other genes. The proportion of an enzyme’s transcripts relative to total transcripts is usually a good indicator of its activity in different cell types regardless of the cell’s total transcriptome size. Thus, for measuring DNG transcript abundance in Figure 3B, we use conventional TPM normalization.

For the timecourse in Figure 3A, the normalized transcript abundances were also smoothed using a weighted average to suppress sample-to-sample noise. The average was performed using a guassian kernel with the sample-specific x-coordinates given as the sample “pseudotime” value as previously defined (Nelms and Walbot 2022). This creates a weighted average where two samples that are more similar in overall expression (e.g. similar pseudotime values) are given more weight than samples that are distinct. The effect of this smoothing is similar to taking the mean expression value by stage, but allows more continuous time resolution without requiring sharp stage boundaries (e.g. a gene that goes up within a stage could be recognized). The heatmap in Supplemental Figure 6 shows the same data without any smoothing.

## DATA ACCESSIBILTY

All raw sequencing data generated in this study are being submitted to the NCBI BioProject database (https://www.ncbi.nlm.nih.gov/bioproject/) under accession number PRJNA1035166. https://github.com/dawelab/MethylatedPollenGenes

## Supplementary Material

Supplement 1

Supplement 2

Supplement 3

Supplement 4

## References

[R1] Adhikari PB, Liu X, Kasahara RD. 2020. Mechanics of Pollen Tube Elongation: A Perspective. Front Plant Sci 11: 589712. 33193543 10.3389/fpls.2020.589712 PMC7606272

[R2] Ando A, Kirkbride RC, Qiao H, Chen ZJ. 2023. Endosperm and Maternal-specific expression of EIN2 in the endosperm affects endosperm cellularization and seed size in Arabidopsis. Genetics 223. 10.1093/genetics/iyac161 PMC9910398 36282525

[R3] Baile F, Gómez-Zambrano Á, Calonje M. 2022. Roles of Polycomb complexes in regulating gene expression and chromatin structure in plants. Plant Commun 3: 100267. 35059633 10.1016/j.xplc.2021.100267 PMC8760139

[R4] Becker JD, Takeda S, Borges F, Dolan L, Feijó JA. 2014. Transcriptional profiling of Arabidopsis root hairs and pollen defines an apical cell growth signature. BMC Plant Biol 14: 197. 25080170 10.1186/s12870-014-0197-3 PMC4236730

[R5] Belele CL, Sidorenko L, Stam M, Bader R, Arteaga-Vazquez MA, Chandler VL. 2013. Specific tandem repeats are sufficient for paramutation-induced trans-generational silencing. PLoS Genet 9: e1003773. 24146624 10.1371/journal.pgen.1003773 PMC3798267

[R6] Borg M, Papareddy RK, Dombey R, Axelsson E, Nodine MD, Twell D, Berger F. 2021. Epigenetic reprogramming rewires transcription during the alternation of generations in Arabidopsis. Elife 10. 10.7554/eLife.61894 PMC7920552 33491647

[R7] Calarco JP, Borges F, Donoghue MT, Van Ex F, Jullien PE, Lopes T, Gardner R, Berger F, Feijó JA, Becker JD 2012. Reprogramming of DNA methylation in pollen guides epigenetic inheritance via small RNA. Cell 151: 194–205. 23000270 10.1016/j.cell.2012.09.001 PMC3697483

[R8] Chen S, Zhou Y, Chen Y, Gu J. 2018. fastp: an ultra-fast all-in-one FASTQ preprocessor. Bioinformatics 34: i884–i890. 30423086 10.1093/bioinformatics/bty560 PMC6129281

[R9] Chen Z, Zhang Z, Zhang H, Li K, Cai D, Zhao L, Liu J, Chen H. 2022. A pair of non-Mendelian genes at the Ga2 locus confer unilateral cross-incompatibility in maize. Nat Commun 13: 1993. 35422051 10.1038/s41467-022-29729-z PMC9010485

[R10] Choi Y, Gehring M, Johnson L, Hannon M, Harada JJ, Goldberg RB, Jacobsen SE, Fischer RL. 2002. DEMETER, a DNA glycosylase domain protein, is required for endosperm gene imprinting and seed viability in arabidopsis. Cell 110: 33–42. 12150995 10.1016/s0092-8674(02)00807-3

[R11] Costa M, Nobre MS, Becker JD, Masiero S, Amorim MI, Pereira LG, Coimbra S. 2013. Expression-based and co-localization detection of arabinogalactan protein 6 and arabinogalactan protein 11 interactors in Arabidopsis pollen and pollen tubes. BMC Plant Biol 13: 7. 23297674 10.1186/1471-2229-13-7 PMC3546934

[R12] Crisp PA, Marand AP, Noshay JM, Zhou P, Lu Z, Schmitz RJ, Springer NM. 2020. Stable unmethylated DNA demarcates expressed genes and their cis-regulatory space in plant genomes. Proc Natl Acad Sci U S A 117: 23991–24000. 32879011 10.1073/pnas.2010250117 PMC7519222

[R13] Flores-Tornero M, Wang L, Potěšil D, Hafidh S, Vogler F, Zdráhal Z, Honys D, Sprunck S, Dresselhaus T. 2021. Comparative analyses of angiosperm secretomes identify apoplastic pollen tube functions and novel secreted peptides. Plant Reprod 34: 47–60. 33258014 10.1007/s00497-020-00399-5 PMC7902602

[R14] Gehring M, Huh JH, Hsieh TF, Penterman J, Choi Y, Harada JJ, Goldberg RB, Fischer RL. 2006. DEMETER DNA glycosylase establishes MEDEA polycomb gene self-imprinting by allele-specific demethylation. Cell 124: 495–506. 16469697 10.1016/j.cell.2005.12.034 PMC4106368

[R15] Gent JI, Higgins KM, Swentowsky KW, Fu FF, Zeng Y, Kim DW, Dawe RK, Springer NM, Anderson SN. 2022. The maize gene maternal derepression of r1 encodes a DNA glycosylase that demethylates DNA and reduces siRNA expression in the endosperm. Plant Cell 34: 3685–3701. 35775949 10.1093/plcell/koac199 PMC9516051

[R16] Greenberg MVC, Bourc'his D. 2019. The diverse roles of DNA methylation in mammalian development and disease. Nat Rev Mol Cell Biol 20: 590–607. 31399642 10.1038/s41580-019-0159-6

[R17] Hafidh S, Honys D. 2021. Reproduction Multitasking: The Male Gametophyte. Annu Rev Plant Biol 72: 581–614. 33900787 10.1146/annurev-arplant-080620-021907

[R18] Halter T, Wang J, Amesefe D, Lastrucci E, Charvin M, Singla Rastogi M, Navarro L. 2021. The Arabidopsis active demethylase ROS1. Elife 10. 10.7554/eLife.62994 PMC7880685 33470193

[R19] Haun WJ, Laoueillé-Duprat S, O'connell MJ, Spillane C, Grossniklaus U, Phillips AR, Kaeppler SM, Springer NM. 2007. Genomic imprinting, methylation and molecular evolution of maize Enhancer of zeste (Mez) homologs. Plant J 49: 325–337. 17181776 10.1111/j.1365-313X.2006.02965.x

[R20] He S, Vickers M, Zhang J, Feng X. 2019. Natural depletion of histone H1 in sex cells causes DNA demethylation, heterochromatin decondensation and transposon activation. Elife 8. 10.7554/eLife.42530 PMC6594752 31135340

[R21] Hemenway EA, Gehring M. 2023. Epigenetic Regulation During Plant Development and the Capacity for Epigenetic Memory. Annu Rev Plant Biol 74: 87–109. 36854474 10.1146/annurev-arplant-070122-025047 PMC10280588

[R22] Henderson IR, Jacobsen SE. 2008. Tandem repeats upstream of the Arabidopsis endogene SDC recruit non-CG DNA methylation and initiate siRNA spreading. Genes Dev 22: 1597–1606. 18559476 10.1101/gad.1667808 PMC2428058

[R23] Hermon P, Srilunchang KO, Zou J, Dresselhaus T, Danilevskaya ON. 2007. Activation of the imprinted Polycomb Group Fie1 gene in maize endosperm requires demethylation of the maternal allele. Plant Mol Biol 64: 387–395. 17437065 10.1007/s11103-007-9160-0

[R24] Hsieh PH, He S, Buttress T, Gao H, Couchman M, Fischer RL, Zilberman D, Feng X. 2016. Arabidopsis male sexual lineage exhibits more robust maintenance of CG methylation than somatic tissues. Proc Natl Acad Sci U S A 113: 15132–15137. 27956643 10.1073/pnas.1619074114 PMC5206529

[R25] Hufford MB, Seetharam AS, Woodhouse MR, Chougule KM, Ou S, Liu J, Ricci WA, Guo T, Olson A, Qiu Y 2021. De novo assembly, annotation, and comparative analysis of 26 diverse maize genomes. Science 373: 655–662. 34353948 10.1126/science.abg5289 PMC8733867

[R26] Ibarra CA, Feng X, Schoft VK, Hsieh TF, Uzawa R, Rodrigues JA, Zemach A, Chumak N, Machlicova A, Nishimura T 2012. Active DNA demethylation in plant companion cells reinforces transposon methylation in gametes. Science 337: 1360–1364. 22984074 10.1126/science.1224839 PMC4034762

[R27] Ichino L, Picard CL, Yun J, Chotai M, Wang S, Lin EK, Papareddy RK, Xue Y, Jacobsen SE. 2022. Single-nucleus RNA-seq reveals that MBD5, MBD6, and SILENZIO maintain silencing in the vegetative cell of developing pollen. Cell Rep 41: 111699. 36417865 10.1016/j.celrep.2022.111699 PMC9770095

[R28] Irshad F, Li C, Wu HY, Yan Y, Xu JH. 2022. The Function of DNA Demethylase Gene ROS1a Null Mutant on Seed Development in Rice (Int J Mol Sci 23. 10.3390/ijms23126357 PMC9223687 35742811

[R29] Jackson SL. 2001. Do hyphae pulse as they grow? New Phytol 151: 556–560. 33853260 10.1046/j.0028-646x.2001.00228.x

[R30] Jia QS, Zhu J, Xu XF, Lou Y, Zhang ZL, Zhang ZP, Yang ZN. 2015. Arabidopsis AT-hook protein TEK positively regulates the expression of arabinogalactan proteins for Nexine formation. Mol Plant 8: 251–260. 25616387 10.1016/j.molp.2014.10.001

[R31] Jia Y, Lisch DR, Ohtsu K, Scanlon MJ, Nettleton D, Schnable PS. 2009. Loss of RNA-dependent RNA polymerase 2 (RDR2) function causes widespread and unexpected changes in the expression of transposons, genes, and 24-nt small RNAs. PLoS Genet 5: e1000737. 19936292 10.1371/journal.pgen.1000737 PMC2774947

[R32] Kende H, Bradford K, Brummell D, Cho HT, Cosgrove D, Fleming A, Gehring C, Lee Y, McQueen-Mason S, Rose J 2004. Nomenclature for members of the expansin superfamily of genes and proteins. Plant Mol Biol 55: 311–314. 15604683 10.1007/s11103-004-0158-6

[R33] Khouider S, Borges F, LeBlanc C, Ungru A, Schnittger A, Martienssen R, Colot V, Bouyer D. 2021. Male fertility in Arabidopsis requires active DNA demethylation of genes that control pollen tube function. Nat Commun 12: 410. 33462227 10.1038/s41467-020-20606-1 PMC7813888

[R34] Kim D, Paggi JM, Park C, Bennett C, Salzberg SL. 2019a. Graph-based genome alignment and genotyping with HISAT2 and HISAT-genotype. Nat Biotechnol 37: 907–915. 31375807 10.1038/s41587-019-0201-4 PMC7605509

[R35] Kim MY, Ono A, Scholten S, Kinoshita T, Zilberman D, Okamoto T, Fischer RL. 2019b. DNA demethylation by ROS1a in rice vegetative cells promotes methylation in sperm. Proc Natl Acad Sci U S A. 10.1073/pnas.1821435116 PMC6511055 31000601

[R36] Kinoshita Y, Saze H, Kinoshita T, Miura A, Soppe WJ, Koornneef M, Kakutani T. 2007. Control of FWA gene silencing in Arabidopsis thaliana by SINE-related direct repeats. Plant J 49: 38–45. 17144899 10.1111/j.1365-313X.2006.02936.x

[R37] Kovaka S, Zimin AV, Pertea GM, Razaghi R, Salzberg SL, Pertea M. 2019. Transcriptome assembly from long-read RNA-seq alignments with StringTie2. Genome Biol 20: 278. 31842956 10.1186/s13059-019-1910-1 PMC6912988

[R38] Love MI, Huber W, Anders S. 2014. Moderated estimation of fold change and dispersion for RNA-seq data with DESeq2. Genome Biol 15: 550. 25516281 10.1186/s13059-014-0550-8 PMC4302049

[R39] Martinez G, Panda K, Köhler C, Slotkin RK. 2016. Silencing in sperm cells is directed by RNA movement from the surrounding nurse cell. Nat Plants 2: 16030. 27249563 10.1038/nplants.2016.30

[R40] Miller EC. 1919. Development of the pistillate spikelet and fertilization in Zea mays L. Journal of Agricultural Research 18: 12

[R41] Muyle AM, Seymour DK, Lv Y, Huettel B, Gaut BS. 2022. Gene Body Methylation in Plants: Mechanisms, Functions, and Important Implications for Understanding Evolutionary Processes. Genome Biol Evol 14. 10.1093/gbe/evac038 PMC8995044 35298639

[R42] Nelms B, Walbot V. 2022. Gametophyte genome activation occurs at pollen mitosis I in maize. Science 375: 424–429. 35084965 10.1126/science.abl7392

[R43] Park K, Kim MY, Vickers M, Park JS, Hyun Y, Okamoto T, Zilberman D, Fischer RL, Feng X, Choi Y 2016. DNA demethylation is initiated in the central cells of Arabidopsis and rice. Proc Natl Acad Sci U S A 113: 15138–15143. 27956642 10.1073/pnas.1619047114 PMC5206524

[R44] Phillips AR, Evans MM. 2011. Analysis of stunter1, a maize mutant with reduced gametophyte size and maternal effects on seed development. Genetics 187: 1085–1097. 21270392 10.1534/genetics.110.125286 PMC3070518

[R45] Quinlan AR, Hall IM. 2010. BEDTools: a flexible suite of utilities for comparing genomic features. Bioinformatics 26: 841–842. 20110278 10.1093/bioinformatics/btq033 PMC2832824

[R46] Rhoades MM, Rhoades VH. 1939. Genetic Studies with Factors in the Tenth Chromosome in Maize. Genetics 24: 302–314. 17246924 10.1093/genetics/24.2.302 PMC1209040

[R47] Rodrigues JA, Hsieh PH, Ruan D, Nishimura T, Sharma MK, Sharma R, Ye X, Nguyen ND, Nijjar S, Ronald PC 2021. Divergence among rice cultivars reveals roles for transposition and epimutation in ongoing evolution of genomic imprinting. Proc Natl Acad Sci U S A 118. 10.1073/pnas.2104445118 PMC8307775 34272287

[R48] Schmitz RJ, Schultz MD, Urich MA, Nery JR, Pelizzola M, Libiger O, Alix A, McCosh RB, Chen H, Schork NJ 2013. Patterns of population epigenomic diversity. Nature 495: 193–198. 23467092 10.1038/nature11968 PMC3798000

[R49] Schoft VK, Chumak N, Choi Y, Hannon M, Garcia-Aguilar M, Machlicova A, Slusarz L, Mosiolek M, Park JS, Park GT 2011. Function of the DEMETER DNA glycosylase in the Arabidopsis thaliana male gametophyte. Proc Natl Acad Sci U S A 108: 8042–8047. 21518889 10.1073/pnas.1105117108 PMC3093457

[R50] Shi J, Cui M, Yang L, Kim YJ, Zhang D. 2015. Genetic and Biochemical Mechanisms of Pollen Wall Development. Trends Plant Sci 20: 741–753. 26442683 10.1016/j.tplants.2015.07.010

[R51] Singleton WR, Mangelsdorf PC. 1940. Gametic Lethals on the Fourth Chromosome of Maize. Genetics 25: 366–390. 17246976 10.1093/genetics/25.4.366 PMC1209097

[R52] Slotkin RK, Vaughn M, Borges F, Tanurdzić M, Becker JD, Feijó JA, Martienssen RA. 2009. Epigenetic reprogramming and small RNA silencing of transposable elements in pollen. Cell 136: 461–472. 19203581 10.1016/j.cell.2008.12.038 PMC2661848

[R53] Smith T, Heger A, Sudbery I. 2017. UMI-tools: modeling sequencing errors in Unique Molecular Identifiers to improve quantification accuracy. Genome Res 27: 491–499. 28100584 10.1101/gr.209601.116 PMC5340976

[R54] Springer NM, Anderson SN, Andorf CM, Ahern KR, Bai F, Barad O, Barbazuk WB, Bass HW, Baruch K, Ben-Zvi G 2018. The maize W22 genome provides a foundation for functional genomics and transposon biology. Nat Genet 50: 1282–1288. 30061736 10.1038/s41588-018-0158-0

[R55] Strader L, Weijers D, Wagner D. 2022. Plant transcription factors - being in the right place with the right company. Curr Opin Plant Biol 65: 102136. 34856504 10.1016/j.pbi.2021.102136 PMC8844091

[R56] Suetsugu N, Wada M. 2016. Evolution of the Cp-Actin-based Motility System of Chloroplasts in Green Plants. Front Plant Sci 7: 561. 27200035 10.3389/fpls.2016.00561 PMC4853393

[R57] Thorvaldsdóttir H, Robinson JT, Mesirov JP. 2013. Integrative Genomics Viewer (IGV): high-performance genomics data visualization and exploration. Brief Bioinform 14: 178–192. 22517427 10.1093/bib/bbs017 PMC3603213

[R58] Tonosaki K, Ono A, Kunisada M, Nishino M, Nagata H, Sakamoto S, Kijima ST, Furuumi H, Nonomura KI, Sato Y 2021. Mutation of the imprinted gene OsEMF2a induces autonomous endosperm development and delayed cellularization in rice. Plant Cell 33: 85–103. 33751094 10.1093/plcell/koaa006 PMC8136911

[R59] Walker EL. 1998. Paramutation of the r1 locus of maize is associated with increased cytosine methylation. Genetics 148: 1973–1981. 9560410 10.1093/genetics/148.4.1973 PMC1460097

[R60] Warman C, Panda K, Vejlupkova Z, Hokin S, Unger-Wallace E, Cole RA, Chettoor AM, Jiang D, Vollbrecht E, Evans MMS 2020. High expression in maize pollen correlates with genetic contributions to pollen fitness as well as with coordinated transcription from neighboring transposable elements. PLoS Genet 16: e1008462. 32236090 10.1371/journal.pgen.1008462 PMC7112179

[R61] Washburn M, Alaniz-Fabián J, Scroggs T, Nelms B. 2023. Single-cell RNA-seq of maize meiocytes and pollen grains. Nat Protoc 18: 3512–3533. 37783945 10.1038/s41596-023-00889-6

[R62] Williams JH, Edwards JA, Ramsey AJ. 2016. Economy, efficiency, and the evolution of pollen tube growth rates. Am J Bot 103: 471–483. 26936897 10.3732/ajb.1500264

[R63] Xu Q, Wu L, Luo Z, Zhang M, Lai J, Li L, Springer NM, Li Q. 2022. DNA demethylation affects imprinted gene expression in maize endosperm. Genome Biol 23: 77. 35264226 10.1186/s13059-022-02641-x PMC8905802

[R64] Yang W, Yao D, Duan H, Zhang J, Cai Y, Lan C, Zhao B, Mei Y, Zheng Y, Yang E 2023. VAMP726 from maize and Arabidopsis confers pollen resistance to heat and UV radiation by influencing lignin content of sporopollenin. Plant Commun 4: 100682. 37691288 10.1016/j.xplc.2023.100682 PMC10721520

[R65] Yang Y, Yu Y, Liang Y, Anderson CT, Cao J. 2018. A Profusion of Molecular Scissors for Pectins: Classification, Expression, and Functions of Plant Polygalacturonases. Front Plant Sci 9: 1208. 30154820 10.3389/fpls.2018.01208 PMC6102391

[R66] Zeng Y, Dawe RK, Gent JI. 2023. Natural methylation epialleles correlate with gene expression in maize. Genetics 225. 10.1093/genetics/iyad146 PMC10550312 37556604

[R67] Zhou LZ, Wang L, Chen X, Ge Z, Mergner J, Li X, Küster B, Längst G, Qu LJ, Dresselhaus T. 2023. The RALF signaling pathway regulates cell wall integrity during pollen tube growth in maize. Plant Cell. 10.1093/plcell/koad324 PMC11062432 38142229

[R68] Zhou S, Li X, Liu Q, Zhao Y, Jiang W, Wu A, Zhou DX. 2021. DNA demethylases remodel DNA methylation in rice gametes and zygote and are required for reproduction. Mol Plant 14: 15. 10.1016/j.molp.2021.06.006 34116223

